# Degeneration in Arousal Neurons in Chronic Sleep Disruption Modeling Sleep Apnea

**DOI:** 10.3389/fneur.2015.00109

**Published:** 2015-05-26

**Authors:** Yan Zhu, Polina Fenik, Guanxia Zhan, Ryan Xin, Sigrid C. Veasey

**Affiliations:** ^1^Center for Sleep and Circadian Neurobiology, Department of Medicine, Perelman School of Medicine, University of Pennsylvania, Philadelphia, PA, USA

**Keywords:** sleep fragmentation, wakefulness, neurodegeneration and neural differentiation, norepinephrine, hypocretins/orexins

## Abstract

Chronic sleep disruption (CSD) is a cardinal feature of sleep apnea that predicts impaired wakefulness. Despite effective treatment of apneas and sleep disruption, patients with sleep apnea may have persistent somnolence. Lasting wake disturbances in treated sleep apnea raise the possibility that CSD may induce sufficient degeneration in wake-activated neurons (WAN) to cause irreversible wake impairments. Implementing a stereological approach in a murine model of CSD, we found reduced neuronal counts in representative WAN groups, locus coeruleus (LC) and orexinergic neurons, reduced by 50 and 25%, respectively. Mice exposed to CSD showed shortened sleep latencies lasting at least 4 weeks into recovery from CSD. As CSD results in frequent activation of WAN, we hypothesized that CSD promotes mitochondrial metabolic stress in WAN. In support, CSD increased lipofuscin within select WAN. Further, examining the LC as a representative WAN nucleus, we observed increased mitochondrial protein acetylation and down-regulation of anti-oxidant enzyme and brain-derived neurotrophic factor mRNA. Remarkably, CSD markedly increased tumor necrosis factor-alpha within WAN, and not in adjacent neurons or glia. Thus, CSD, as observed in sleep apnea, results in a composite of lasting wake impairments, loss of select neurons, a pro-inflammatory, pro-oxidative mitochondrial stress response in WAN, consistent with a degenerative process with behavioral consequences.

## Introduction

All individuals with untreated obstructive sleep apnea experience chronic sleep disruption (CSD), and this feature of sleep apnea is considered an important source of impaired wakefulness (1, 2). While most patients successfully treated have marked improvements in hypersomnolence, many effectively treated individuals experience refractory or persistent wake impairments (3). The mechanisms by which hypersomnolence persists in treated apnea are not known. Plausible explanations include metabolic derangements, particularly in obese patients, depression, and additional sleep disorders. An alternative explanation for residual sleepiness in treated sleep apnea is that CSD might injure neurons responsible for optimal alertness sufficiently enough to manifest as lasting wake impairments.

Diverse neuronal groups, and potentially glia, influence sleep and wakefulness (4–6). Within this complex circuitry are subsets of neurons that are activated upon arousal and in turn modulate wakefulness. These wake-activated neurons (WAN) groups include the noradrenergic locus coeruleus (LC), orexinergic, histaminergic neurons, and also specific cholinergic, serotoninergic, and dopaminergic nuclei. While sufficient loss of orexinergic neurons causes narcolepsy, loss of one or more of the other wake modulatory groups has little effect on total wake time (7), yet may have profound effects on attention and vigilance and the ability to sustain wakefulness (8, 9). Thus, it is anticipated that injury to these wake modulatory WAN would impair aspects of wakefulness.

Wake-activated neurons typically project extensive collaterals and produce large quantities of neuromodulators and/or neurotransmitters (10, 11). Thus, the neurons have high metabolic demands under normal conditions, and are susceptible to metabolic stress upon increased activation or metabolic perturbances (12–14). We recently found that adult mice exposed to 4 weeks of CSD developed impaired arousal responses and blunted WAN activation responses that might contribute to arousal responses (15). To extend these findings, we sought to determine first whether CSD could result in degeneration of WAN, impart lasting wake impairments and to then explore mechanisms of injury. Having recently identified mitochondrial metabolic stress as a source of injury in chronic short sleep (16), we determined whether CSD would also manifest as mitochondrial metabolic stress in WAN. Tumor necrosis factor-alpha (TNF-α) is up-regulated in the brains of animals’ models of sleep fragmentation and short sleep (17, 18). The source of TNF-α is presumed to be glial, yet this has not been confirmed, and there are reports of neuronal TNF-α (19, 20). In the present studies, we examined TNF-α responses in WAN nuclei. A complement of electrophysiologic, neuroanatomic, and molecular approaches was implemented to characterize WAN metabolic and inflammatory responses to CSD and the lasting consequences of CSD on wakefulness and wake neuron survival.

## Materials and Methods

### Mice and study overview

Studies were performed at the University of Pennsylvania with the approval of the Institutional Animal Care and Use Committee and in accordance with the revised National Institutes of Health Office Laboratory Animal Welfare Policy. C57/BL6 male mice (Jackson Laboratory) were 8 weeks of age at the start of experimentation. Four B6.129S-Tnftm1Gkl/J (TNF-α^−/−^) male mice were used as negative controls in TNF-α immunohistochemical studies. Studied mice were born in our colony and were continually housed in a light/dark environment (lights-on from 6 a.m. to 6 p.m.) and fed *ad libitum* standard rodent chow and water. Ambient temperature and humidity were maintained between 22–24°C and 40–60%, respectively.

### Chronic sleep disruption protocol

Mice were randomized to receive 14 weeks of CSD or control (Ctl) conditions. CSD was performed across the entire 24 h period to ensure total sleep fragmentation (as observed in sleep apnea) and to avoid having the mice increase sleep during their habitual active period, thereby changing their circadian rhythms. Previously, we have shown that total sleep time and sleep distribution across 24 h are unchanged in this paradigm, while arousal frequency is doubled (15). CSD was induced using methods designed and initially validated by Sinton et al. (21) using an orbital rotor (MaxQ 2000; Thermo Scientific, Marietta, OH, USA) with speed set at 110 rpm, and a repeated cycle of 10 s-on, 50 s-off continuously across 14 weeks controlled by a timer (H3CR-F8-300, OMRON Corporation, Kyoto, Japan). An auditory stimulus used in the Sinton model was omitted to avoid awakening Ctl mice. A target arousal frequency of 60/h was chosen to double the arousal frequency in mice. Ctl mice were exposed to 2 h of rotor motion continuously for the first 2 h of the dark period to experience a similar number of rotations/day, yet to avoid sleep disruption. Standard mouse cages were placed on enlarged rotor platforms (65 cm × 120 cm). Mice under CSD and Ctl conditions were able to groom, eat, and drink during orbital rotor movement of the platform. Water bottles with long nozzles equipped with ball valve tips were used to prevent leakage with platform movement. Previously, we established that mice maintained in these conditions over prolonged periods gain weight normally and do not manifest increased plasma corticosteroid levels (15).

#### Surgery and Behavioral State Recording and Analysis

A subset of mice was examined for sleep/wake effects following 14 weeks CSD and a 4-week recovery period (*n* = 8) or Ctl conditions (*n* = 7). For chronic sleep recording electrode implantation, mice were removed from the CSD and Ctl conditions for 2 weeks and then anesthetized with ketamine (90–100 mg/kg) and xylazine (10 mg/kg) and implanted as previously described with fronto-cortical electroencephalographic (EEG), occipital ground, and dorsal nuchal electromyographic (EMG) recording wires and electrode connector (22). Following a 1-week post-operative period, mice were connected to a counterweighted recording cable. At 4 weeks into recovery from CSD or Ctl conditions, electrographic signals were amplified, digitized, and recorded and exported into the SleepSign sleep/wake program (version 3.0, Kissei) for analysis. Wake–sleep states were scored in 4 s epochs, using recently detailed criteria for state determination. As CSD effects on sleep parameters have recently been described, including use of our paradigm (15, 23), we focused the present analysis on wake parameters: 24 h total wake time and hourly distribution and latencies to sleep in the active period (dark onset period) and sleep-predominant (lights-on) period. Data were analyzed using one- and two-way ANOVA, corrected for multiple comparisons using Sidak’s for the three behavioral states.

#### Immunohistochemistry

Histological studies were performed for optical fractionator stereological counts and for characterization of WAN injury. Mice exposed to CSD and Ctl conditions (*n* = 5–6/group, all after the 4-week recovery) were anesthetized with pentobarbital for transcardial perfusion with 4% paraformaldehyde. Post-fixed, cryoprotected brains were sectioned coronally at 60 μm for 1:6 section series of the complete brain (24). From the 1:6 series, a 1:2 rostral–caudal complete set of orexinergic and a 1:3 set of noradrenergic LC neurons were selected for optical fractionator stereological counts. Primary antibodies details and titers were recently reported (24). Visualization of target antigens was performed using Vector Blue labeling (Vector Laboratories) of orexin or tyrosine hydroxylase (TH) immunolabeled neurons and counterstained with Giemsa for detection of all neurons and their nuclei within the counting frame. Using a Nikon Eclipse 600 microscope and a Stereo Investigator workstation (MicroBrightField), an optical fractionator approach (25) was validated to estimate the total number of LC and orexinergic neurons per mouse. For LC neuron counts, all Giemsa labeled somata >15 μm diameter within the LC nucleus with a nucleus that came into focus within the counting frame (probe) were counted using a 100× oil objective. A sampling scheme of 0.25 area sampling frequency for LC and 0.16 for orexinergic neurons with a thickness sampling frequency of 0.79 (allowing 2 μm guard zones on either side) were used. In preliminary studies implementing wild-type rested mice, this strategy provides >150 counts/mouse and Gundersen coefficients of error <0.10. For orexinergic neuron number estimates, only orexinergic-labeled neurons with visible nuclei were included. By performing counts after a 4-week recovery period, we reasoned that if short-term down-regulation of orexin in neurons contributes to reduced counts that this would be resolved after a 4-week recovery opportunity. Neuronal counts were averaged across two counters (>90% agreement) and analyzed using one-way ANOVA and Sidak’s multiple comparisons test for the two WAN groups.

Noradrenergic and orexinergic WAN and melanin-concentrating hormone (MCH) sleep-active neurons were examined for lipofuscin analyzed for autofluorescence intensity (excitation 488 nm, emission 550–650 nm) and Alexa Fluor 488 labeling of WAN. Anti-MCH (H-070-47, Phoenix Pharm) at 1:1000 was used to identify MCH neurons. Noradrenergic, orexinergic, MCH, and histaminergic neurons were examined for TNF-α normalized immunointensities. TNF-α was detected using with 3707, Cell Signaling at 1:2000. Histaminergic neurons were targeted for visualization with anti-histidine decarboxylase antibody (B-GP-265-1, Euro-diagnostica, 1:1000). Each neuronal population was visualized with Alexa Fluor 594 and TNF-α with 488 (Invitrogen). Imaging was performed using a SP5/AOBS confocal microscope; for confocal image acquisition, laser intensities and frequencies, exposure time, detector gain, amplifier offset, and depth of the focal plane within the section were standardized to allow comparison across sections (24). Both lipofuscin and TNF-α were analyzed using one-way ANOVA for sleep condition with Sidak’s multiple comparison test for three or four WAN groups.

#### Brain Tissue Procurement, Mitochondrial Isolation, and Westerns

Additional groups of mice (CSD and Ctl groups + recov, *n* = 5–12/group) were used for protein and RNA studies. These mice were anesthetized and transcardially perfused with ice-cold phospho-buffered saline with RNase inhibitor. Brains were rapidly removed and sectioned coronally for tissue punches (1 mm^3^) of LC bilaterally and stored in −70°C. Mitochondria isolation and cytosolic fractionation were performed using a commercial mitochondria isolation kit (#89801, Thermo Scientific) according to manufacturer’s recommendations using their Dounce homogenization protocol. Mitochondria-enriched samples were verified with a mitochondrial-specific protein, voltage-dependent anion channel 1 (VDAC1). Total lysine-acetylated protein were analyzed using our previously published western blot protocol (24) using 9441, Cell Signaling at 1:500. Images were analyzed with Odyssey Application software, version 3.0.16 (Li-Cor), for mean integrated densities that were normalized to VDAC1 and analyzed with unpaired *t*-test for the one variable.

#### Quantitative Real-Time PCR

Quantitative real-time PCR was performed on LC and LH punches to examine mitochondrial sirtuin type 3 (SirT3), TNF-α, brain-derived neurotrophic factor (BDNF, total), catalase, and SOD2 transcriptional responses to short-term sleep loss in CSD and Ctl mice (*n* = 5–10/group) using recently detailed methods (24). Sequences for some of Taqman primer/probe sets, designed using Primer Express 2.0.0 software, are detailed in previous papers: SirT3, SOD2, and catalase (16), TH, dopamine beta-hydroxylase (DBH), and orexin (24). For BDNF (NM_007540), we used sense: GGGTCACAGCGGCAGATAA (1107–1125); anti-sense: TGCAGCCTTCCTTGGTGTAAC (1256–1236); and probe: TCCCGGTATCCAAAGGCCAACT (1176–1197). Primer/probe sets showed excellent sensitivity (detection of >10^4^ copies/sample) and Ct vs. log copies linearity (*r*^2^ > 0.99). Copy numbers were analyzed using two-way ANOVA for gene and sleep condition, with multiple comparisons corrected using Sidak’s–Bonferroni *t*-test.

## Results

### CSD imparts lasting wake impairments, without influencing total sleep time

**Figure 1:**
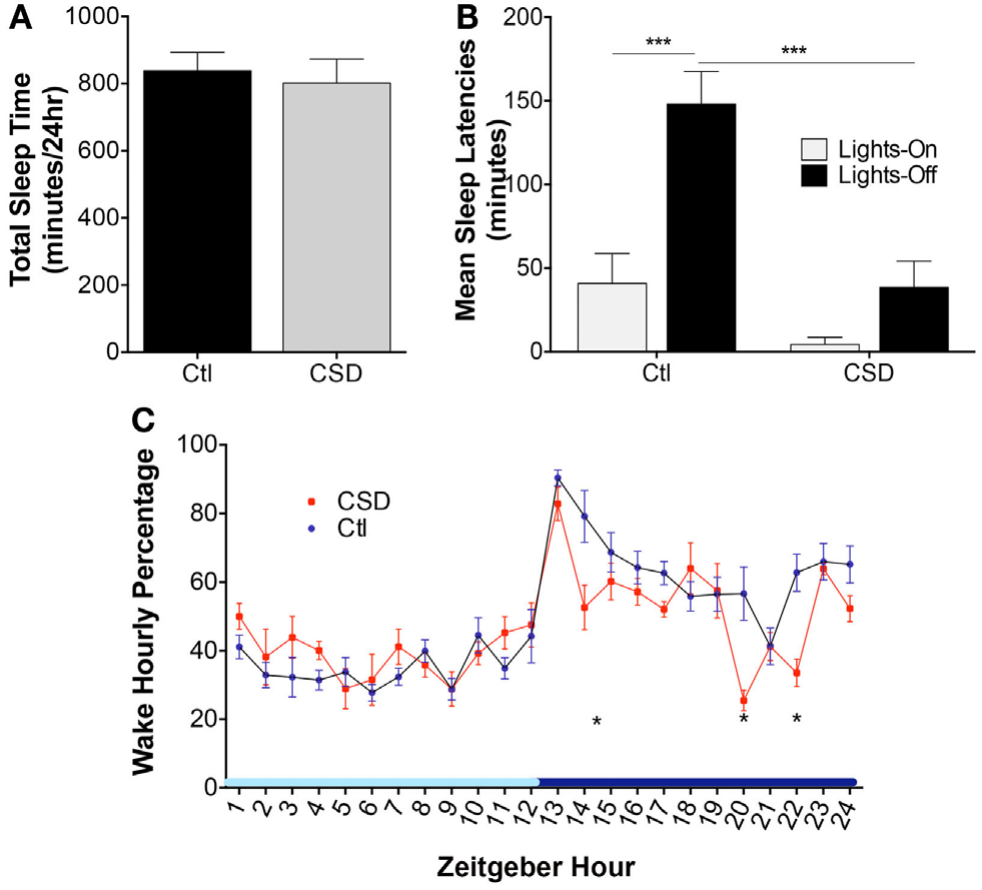
**Effects of rotor platform chronic sleep disruption (CSD) on wakefulness**. **(A)** Group data during CSD for total sleep time across 24 h in control (Ctl, black bar, *n* = 7 mice) and CSD (gray bar, *n* = 8 mice). Data shown are mean ± SE values. **(B)** Group sleep latencies following CSD or Ctl conditions and a 4-week recovery period (normal sleep conditions). Mean ± SE are presented for the lights-on (light gray bars) and lights-off (black bars), ***p < 0.001. **(C)** Hourly percentiles of time spent in wakefulness across the 24-h light–dark cycle, denoted as Zeitgeber time relative to lights-on onset. Data are mean ± SE for CSD (red circles) and Ctl mice (blue circles). *p < 0.05 for multiple *t*-test significances for each hour, as detailed in results. Light blue *x*-axis bar denotes lights-on and dark bar lights-off periods.

There were no differences in total sleep time following CSD and Ctl conditions, *t* = 1.1, N.S. These data are summarized in Figure 1A. Previous studies by multiple groups have highlighted shortened sleep latencies immediately following CSD. Here, we sought to determine whether 4-week recovery time in home cages resting unperturbed is sufficient to fully reverse shortened sleep latencies. Mice exposed to CSD and then allowed a 4-week recovery opportunity were assessed for sleep latencies at lights-on and lights-off. Overall, there were significant CSD and circadian time differences, *F* = 16.5, *p* < 0.0001, as summarized in Figure 1B. Ctl mice demonstrated a longer sleep latency for the lights-off period, relative to lights-on, *t* = 4.9, *p* < 0.001. In contrast, CSD mice showed no difference in sleep latency for the lights-off and -on periods, *t* = 1.5, N.S. There was no effect of CSD on the sleep latency for the lights-on period, *t* = 1.7, N.S.; however, there was a significant reduction in the sleep latency for CSD mice relative to rested for the lights-off period, *t* = 5.0, *p* < 0.001. Examining the hourly distribution of wake time across a 24-h period, there were three times (corrected for 24 comparisons) within the active period that CSD mice showed reduced wake times, as summarized in Figure 1C. In all CSD mice, the wake period at lights-off was significantly shorter. Thus, CSD using this paradigm results in lasting effects on sleep latencies and wake times during the active (lights-off period).

### Wake-activated neuron loss in CSD

**Figure 2:**
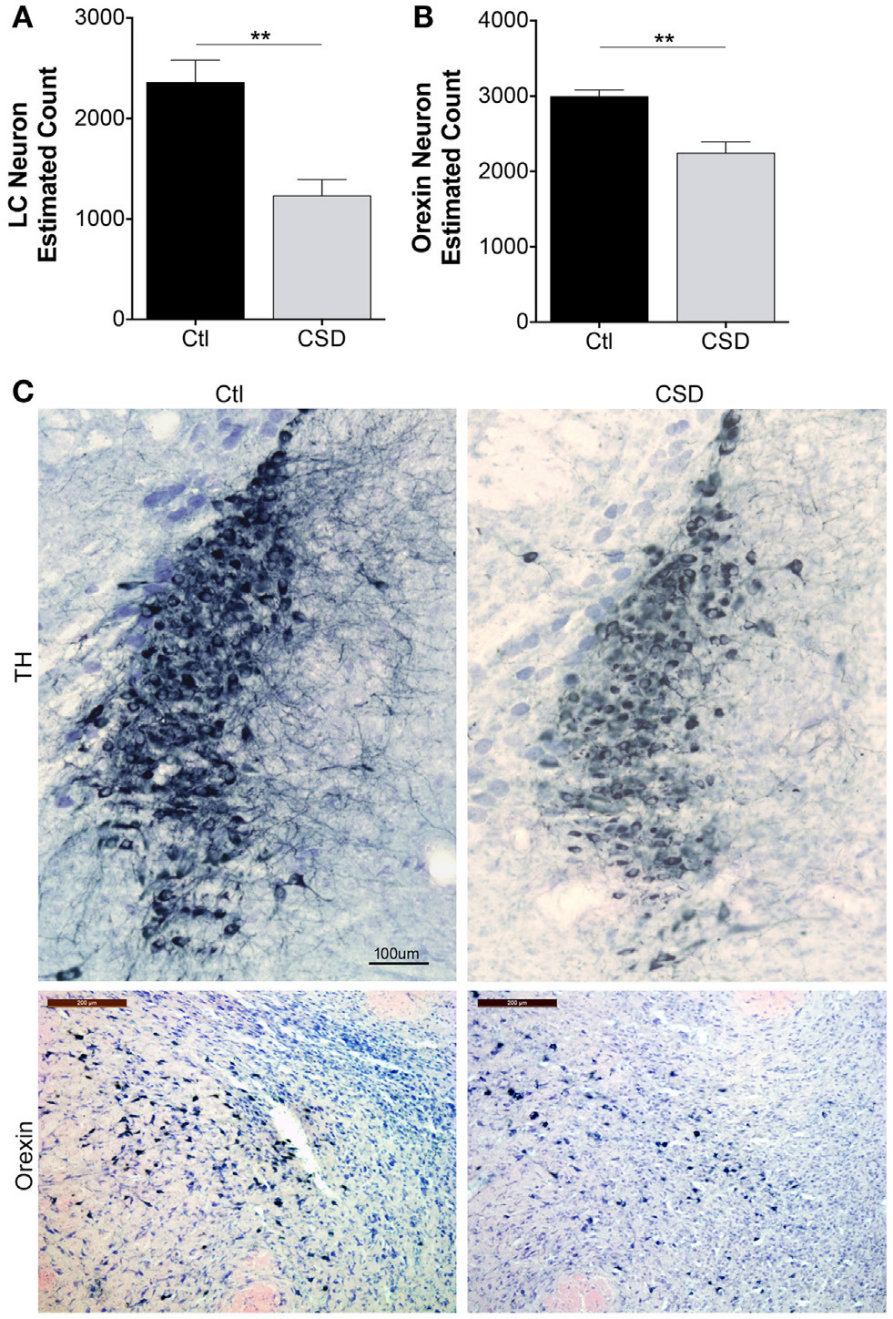
**Reduced neuron estimates for locus coeruleus (LC) and orexinergic neurons in CSD**. **(A)** Optical fractionator stereological bilateral count estimates on 1:3 series of LC sections/mouse for mice exposed to Ctl (black bar) or CSD (gray bar) conditions obtained after a 4-week recovery opportunity for both groups (*n* = 5/group), **p < 0.01. **(B)** A similar stereological approach was used on a 1:2 series of orexinergic neuron sections/mouse for mice exposed to Ctl (black bar) or CSD (gray bar) conditions obtained after a 4-week recovery opportunity for both groups (*n* = 5/group), **p < 0.01. **(C)** Upper panels, representative images from mid LC in a Ctl (left) mouse and CSD mouse (right), immunolabeled with tyrosine hydroxylase (TH) and substrate blue. All TH+ and TH− cells are stained with Giemsa to provide nuclear details for counts and to identify non-TH neurons in LC nuclei. Calibration bar, 100 μm. Lower panels, similarly obtained images of perifornicular orexinergic neurons in Ctl and CSD mice. Calib bar, 200 μm. Note in CSD mice, both LC and orexinergic remaining neuronal somata are significantly smaller.

Previously, we observed reduced numbers of catecholaminergic (TH+) and orexinergic (orexin+) projections to the frontal cortex in mice exposed to CSD (15). Here, we sought to extend findings to LC and orexinergic neuron survival. To exclude the possibility that CSD temporarily down-regulates neuronal markers, e.g., TH for LC and orexin for orexinergic neurons, we performed neuronal counts after 4 weeks recovery from CSD or Ctl, allowing mice to sleep in home cages unperturbed for the 4-week (*n* = 5–6/condition). Overall, there were significant effects of CSD on LC and orexin neuron counts, *F* = 11.4, *p* < 0.001. Relative to Ctl mice, LCn counts were reduced by 50% in CSD, *t* = 4.2, *p* < 0.001 as summarized in Figure 2A. We next examined the effect of CSD on orexinergic neurons and found that CSD resulted in a 25% loss of orexinergic neurons relative to age-matched Ctl mice, *t* = 2.4, *p* < 0.05, Figure 2B. Examination of the remaining neurons for both LC and orexinergic neurons revealed smaller WAN with fewer dendritic projections, consistent with previous findings, as illustrated in Figure 2C. Thus, CSD results in degeneration of LC and orexinergic WAN.

### Metabolic compromise in surviving neurons

**Figure 3:**
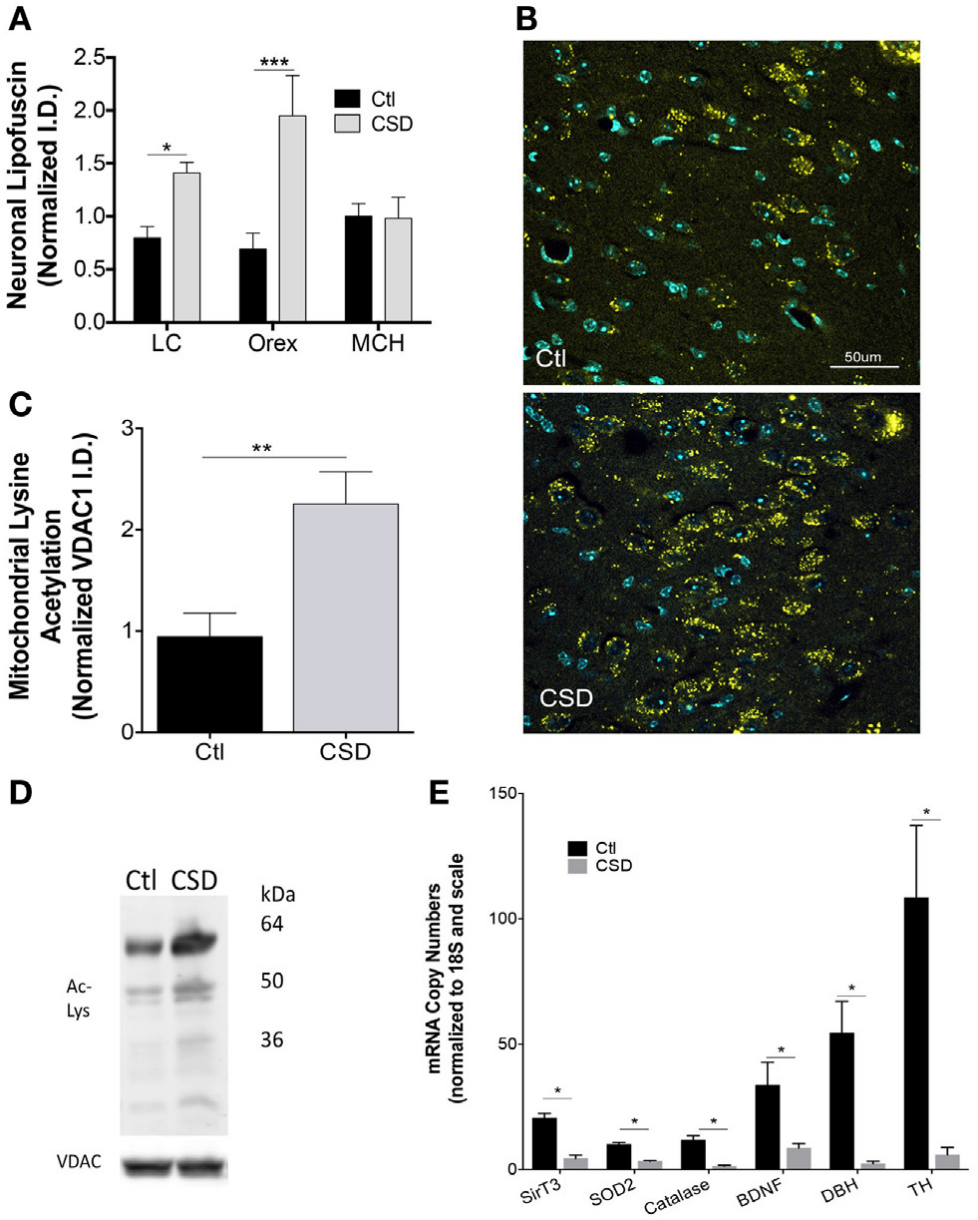
**Metabolic dyshomeostasis in WAN in CSD**. **(A)** Group lipofuscin autofluorescence intensity (excitation 488, emission 550–650) for LC, orexin, and melanin-concentrating hormone (MCH) neurons in mice exposed to Ctl (black bars) and CSD (gray bars). Data are presented as mean ± SE for *n* = 5–7/group, *p < 0.05; ***p < 0.001. **(B)** Representative images of autofluorescence (yellow) in sections from Ctl (upper) and CSD (lower) mice in LC neurons. DAPI (blue) labels nuclei within the sections. TH labeling is omitted to highlight the distribution of lipofuscin with the LC nucleus. **(C)** Group data (mean ± SE) for lysine-acetylated protein (total) in mitochondrial subcellular fractionations from LC nuclei, normalized to mitochondrial protein VDAC1 in Ctl (black bar) and CSD (gray bar) (*n* = 5/group), **p < 0.01. **(D)** Representative immunoblots of LC lysine-acetylated proteins and VDAC1 in a Ctl (left) and CSD (right) mouse. Kilodalton scale is provided for reference. **(E)** Quantitative RT-PCR data (mean ± SE, *n* = 7–12/group) for LC tissue punches from Ctl (black bars) and CSD mice (gray bars). Copy numbers were normalized to ribosomal 18S RNA (loading control) and then to the scale for all genes. *p < 0.05 in a multiple *t*-test analysis.

Having identified mitochondrial metabolic compromise in LC neurons in mice exposed to repeated episodes of short sleep (16), we next examined whether CSD (without sleep curtailment) also imparts metabolic stress in WAN mitochondria. Cardinal features of mitochondrial stress include oxidative stress and hyperacetylation of mitochondrial proteins. Lipofuscin is comprised of, in part, effete mitochondrial debris within lysosomes. The accreted material emits oxygen radicals and is a marker of significant oxidative stress (26). Lipofuscin has autofluorescence allowing detection with fluorescent microscopy in tissue samples processed with little Triton. Lipofuscin was markedly increased in LC neurons of mice exposed to CSD, *t* = 3.0 and *p* < 0.05, as shown in Figure 3A. Similarly lipofuscin increased in orexinergic neurons in response to CSD, *t* = 4.3 and *p* < 0.001, as shown in Figures 3A,B. To determine whether the observed CSD-increase in lipofuscin is specific to WAN, we measured lipofuscin in sleep-active MCH neurons and found no increase in lipofuscin in MCH neurons in mice exposed to CSD, *t* = 0.1, N.S., Figure 3A. Thus, CSD increases lipofuscin in LC and orex WAN without influencing the orexin-adjacent sleep-active group, MCH.

The LC is a compact population of WAN and thus is suitable for tissue punch protein and PCR assays. In contrast, orexinergic neurons comprise a minority of lateral hypothalamic neurons. Thus, we determined the effects of CSD on mitochondrial protein acetylation in LC micropunches. Mitochondrial protein acetylation increased significantly in mice exposed to CSD, *t* = 3.3, *p* < 0.01, Figures 3C,D.

We next measured mRNA responses within the LC nucleus to CSD to characterize the oxidative stress response, finding marked reductions in mitochondrial sirtuin type 3 (SirT3): *t* = 6.7, and mitochondrial anti-oxidants, superoxide dismutase 2 (SOD2): *t* = 6.6, and catalase, *t* = 5.2. BDNF is essential for LC axonal growth in aging and the LC provides BDNF to its forebrain targets. CSD resulted in lasting suppression of BDNF, *t* = 2.7 (Figure 3E). Both TH and DBH are necessary for noradrenaline production in noradrenergic neurons. CSD resulted in robust reductions of both TH: *t* = 4.1 and DBH: *t* = 3.5, all significant at *p* < 0.05 for multiple comparisons, Figure 3E. Thus, CSD reduces mRNA levels for many proteins essential to the metabolic health, growth and maintenance, and neuromodulator synthesis.

### Up-regulation of tumor necrosis factor in wake-activated neurons

**Figure 4:**
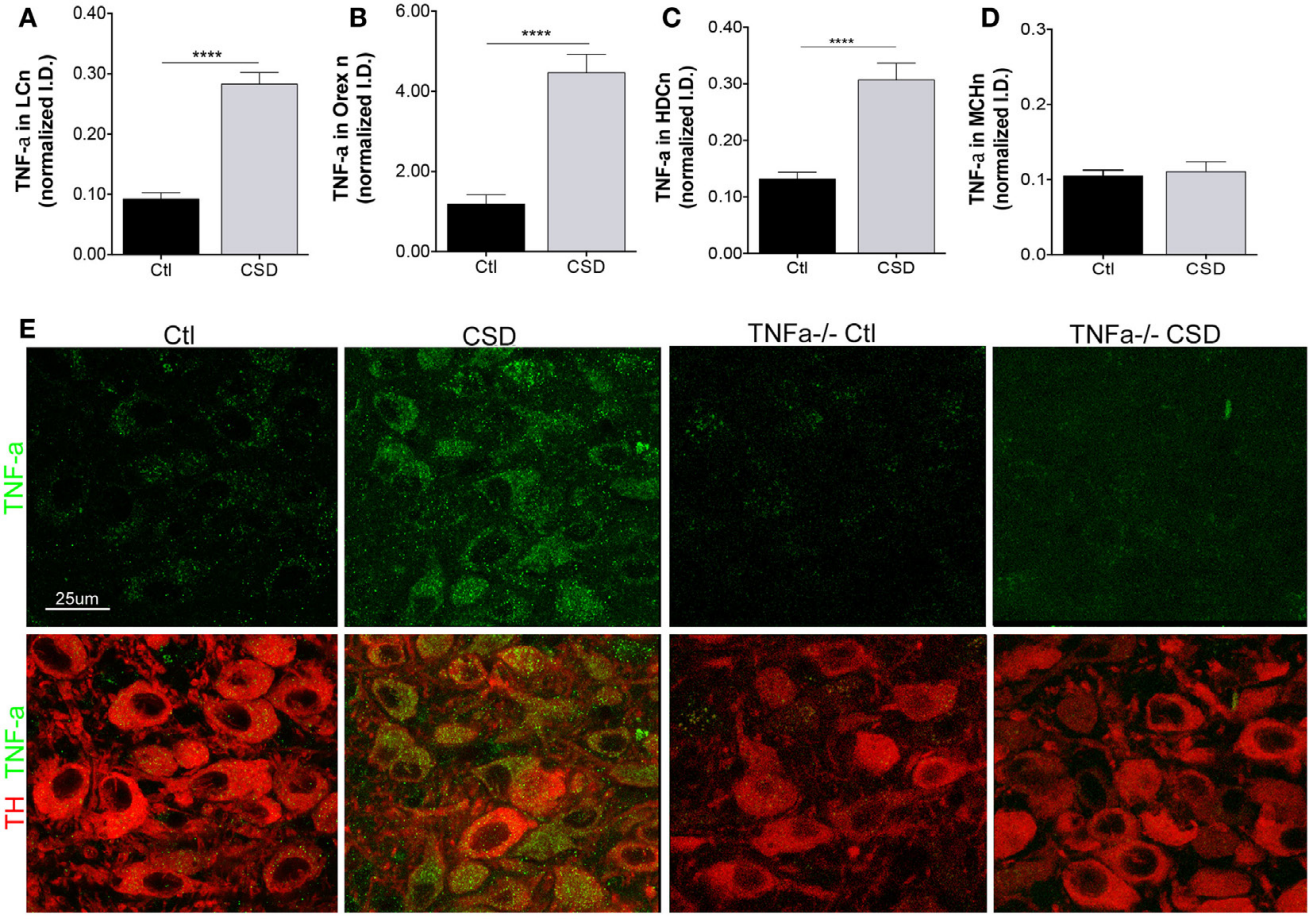
**Tumor necrosis factor-alpha (TNF-α) up-regulation in WAN**. **(A–D)**, TNF-α responses were analyzed for Ctl and CSD mice after the 4-week recovery period. Mean ± SE immunointensity of TNF-α within cell bodies of Ctl mice (black bars) and CSD mice (gray bar) for **(A)**, LC; **(B)**, orexinergic; **(C)** histaminergic tuberomammillary neurons labeled with histidine decarboxylase (HDC); and **(D)**, MCH neurons. ****p < 0.0001. **(E)** Representative images of LC neurons in mice exposed to Ctl and CSD conditions, including TNF-α^−/−^ mice *(right)* to demonstrate antibody specificity. LC neurons are labeled with TH (red) and TNF-α (green).

A strikingly consistent finding across acute and chronic sleep curtailment and short-term and long-term sleep fragmentation is up-regulation of TNF-α in the forebrain (27). We hypothesized that CSD would increase TNF-α in glia adjacent to WAN and that this might contribute to WAN injury. As Breder et al. identified TNF-α in neurons in the healthy mouse in a region consistent with the histaminergic tuberomammillary WAN (20), we included TMN neurons in our analysis. To ensure selectivity of the TNF-α immunoreactivity, we also studied four TNF-α^−/−^ mice, two exposed to CSD and two to Ctl conditions and to determine whether the CSD response is specific WAN, we also examined TNF-α in MCH neurons. CSD resulted in a marked up-regulation of TNF-α within the somata of LCn, *t* = 8.7, *p* < 0.0001, as summarized in Figure 4A. TNF-α was also markedly up-regulated in the somata of orexinergic neurons of mice exposed to CSD, *t* = 6.5, *p* < 0.0001, Figure 4B, and TNF-α was similarly increased in histaminergic TMN neurons, *t* = 5.1, *p* < 0.0001, Figure 4C. In contrast, TNF-α was not increased in MCH neurons of mice exposed to CSD, *t* = 0.4, N.S., Figure 4D. There was minimal TNF-α signal evident in TNF-α^−/−^ mice, as shown in Figure 4E. Thus, CSD results in a substantial increase in TNF-α that appears to be specific to WAN without affecting TNF-α in the MCH sleep-active group of neurons.

## Discussion

In health, wakefulness is determined largely by an overlay of sleep homeostasis on circadian influences on sleep/wake regulatory neuronal groups. This paradigm predicts reversibility in wake impairments, which are not always seen in treated obstructive sleep apnea. The present findings support the concept that long-term intermittent disruption of sleep without affecting total sleep time is sufficient to injure WAN and that this injury may include WAN degeneration and irreversible wake impairments during the intended active period of the circadian rhythm. In remaining LC and orexinergic WAN, CSD imposed significant oxidative stress as evidenced by increasing lipofuscin, while decreasing mitochondrial anti-oxidant enzymes, supporting a maladaptive response to CSD in these neurons. In addition, there was significant mitochondrial metabolic stress as evidenced by increased mitochondrial protein acetylation and reduced SirT3. Remarkably, CSD resulted in a pronounced and lasting increase in TNF-α in the cell bodies of WAN. Collectively, the work provides evidence that sleep consolidation is essential for the health and survival of WAN.

Several methods of sleep fragmentation have proven effective in the ability to chronically disrupt sleep, and each method has its own strengths and limitations. Methodologies that require the animal to ambulate at each intended arousal interval (28) include use of a treadmill (28), a sweeper bar that the animal must climb over (29), and a rotating floor within a cage (30). Each of these is highly effective in inducing arousals at intended intervals. The increased locomotor activity, which is not a feature of CSD in sleep apnea, may increase neuroprotectants in the brain including SirT1 and BDNF (31, 32). The approach developed by Sinton et al. awakens mice through postural movement in sleep (21). This movement is approximately 70–80% effective in inducing arousals for many months. Advantages we perceive with the rotor platform approach include that mice may be group housed in their home cages and have the opportunity to build and preserve their nests. With this paradigm, mice maintain normal circadian sleep/wake patterns, and body weights are unaffected by CSD. In contrasts, models of 12 h CSD across the lights-on period only, mice consistently gain weight (33–35). Despite the diverse CSD paradigms, effects on sleep and sleep latencies are rather consistent, with most studies finding shortened sleep bouts, without reductions in total sleep time, and shortened sleep latencies immediately following CSD (15, 23, 36, 37). We now extend these findings with the observations that shortened sleep latencies and reduced wake times in the lights-off period persist for at least 4 weeks after return to normal uninterrupted sleep; these findings support long-lasting, if not irreversible, wake impairments upon CSD.

In the present study, focusing on two groups of WAN, the noradrenergic LC and the orexinergic (orex) neurons, we found that CSD results in a significant reduction in neuronal counts. Use of an optical fractionator stereological approach excludes the possibility that counts are reduced simply by shrinkage of neurons. By examining counts after a 4-week recovery opportunity, we believe that the reduced counts are consistent with either neuron death or epigenetic re-programing. Our observed reduced transcriptional responses would be consistent with either. However, in the LC nuclei of CSD mice, there were few TH negative neurons to support a significant de-differentiation re-programing. LC and orex neuronal counts decline across aging (38–40), and thus, one possibility is that CSD accentuates or accelerates the aging process within these neurons. In support, we observed increased lipofuscin, a marker of cellular senescence. Further studies including a more comprehensive aging phenotype (telomere shortening, lipid peroxidation, carbonylation, DNA methylation changes, and refined timelines of changes) are needed to discern accelerated or accentuated aging and delayed reversibility.

In the present study, we focused largely on select populations of modulatory WAN; however, it is entirely possible that other neuronal groups and potentially glia are affected by CSD. A human cross-sectional analysis found that CSD in older humans is associated with loss within galanin-positive neurons in the ventrolateral pre-optic area, many of which are sleep-activated neurons and are important in regulating sleep (41). We elected to examine MCH neurons for their proximity to orexinergic neurons to better ascertain whether the effects of CSD on neurons are predicted by cell type or brain region. Although we did not perform stereological counts on MCH neurons, we did not observe an effect on lipofuscin, TNF-α, or morphology. Thus, we anticipate that neurons activated repeatedly upon arousal and those with heightened sensitivity to metabolic disturbances, such as the LC and orexinergic neurons are among subsets of neurons more vulnerable to sleep fragmentation.

Examining metabolic responses of LC neurons to CSD, we observed increased mitochondrial protein acetylation, a marker of impaired SirT3 activity, and mitochondrial metabolic stress. In support, SirT3 mRNA levels were markedly reduced in LC tissue punches. Additional support for CSD-induced metabolic dyshomeostasis in WAN is provided by the increase in lipofuscin and the reductions in transcription levels of mitochondrial anti-oxidant enzymes, superoxide dismutase 2 and catalase, and the observed reduction in BDNF mRNA is also consistent with severe metabolic stress (32). Thus, CSD imparts metabolic stress to WAN while lowering the anti-oxidant response, and in doing so may also render these neurons more susceptible to unrelated physiological challenges, and at the same time less able to rebuild projections.

We anticipated that, in response to CSD, TNF-α would be increased within microglial cells adjacent to WAN, thereby contributing to TNF-α receptor activation-induced neuronal injury (31). Remarkably, TNF-α was robustly up-regulated within the somata of LC, orexinergic, and histaminergic WAN and not within surrounding glia or neurons. Previously, TNF-α immunoreactivity was demonstrated in both neuronal cell bodies and neurites within regions implicated in metabolism and autonomic function (20) and TNF-α can be up-regulated in cortical neurons in an activity-dependent fashion (19). In the present study, very little TNF-α was present in WAN in Ctl mice, while in CSD mice, somatic TNF-α was localized exclusively to WAN within regions examined, the lateral and posterior hypothalamus and dorsal pons, consistent with increased activation in WAN across CSD. The cellular distribution of TNF-α that we observed is quite distinct from the groups of neurons in which TNF-α is up-regulated after exposure to lipopolysaccharide, where neurons in the ventromedial hypothalamus and dorsal medulla show up-regulation of TNF-α mRNA (42). The presence of TNF-α mRNA in neurons supports TNF-α synthesis within neurons rather than uptake, while up-regulation in distinct neuronal in response to CSD or lipopolysaccharide supports the concept that TNF-α neuronal responses vary with specific circuit activation. Mechanisms by which TNF-α may be up-regulated in response to activation or neuronal metabolics and why TNF-α is up-regulated within neurons are not well understood. TNF-α receptors can localize to mitochondria (43), and TNF-α exposure at physiological levels impairs mitochondrial function while increasing the production of reactive oxygen species (44), raising the possibility that neuronal TNF-α serves in intraneuronal signaling rather than as a cell to cell communicator. We observed TNF-α up-regulation well into the recovery period when WAN firing should be normalized, yet oxidative stress remained high, providing a potential source for the persistent TNF-α levels that, in turn, may further injure mitochondria and increase oxidative stress. Such a feed forward cycle might explain the lasting wake impairments and continued injury in remaining WAN observed well into recovery from CSD and deserves further study.

The present collective findings demonstrate that long-term fragmentation of sleep without affecting total sleep time is sufficient to induce lasting wake impairments and impose metabolic stress with mitochondrial dyshomeostasis and degeneration in, at least subsets of, WAN essential for optimal alertness. Clinical implications extend beyond obstructive sleep apnea to other conditions in which sleep is repeatedly briefly interrupted, such as in aging, substance abuse, chronic pain, Parkinson’s disease, Alzheimer’s disease, and restless legs syndrome with periodic limb movements. The present work has focused on select populations of WAN. Future directions should include characterizing the extent of neuronal injury in the brain and identify specific CSD-vulnerable as well as -resistant groups of neurons to facilitate elucidation of mechanisms. A longer recovery opportunity may be necessary to reverse metabolic and behavioral consequences. Additionally, the relative roles of mitochondrial stress and WAN TNF-α in WAN injury and degeneration should be determined to open therapeutic avenues.

## Conflict of Interest Statement

The authors declare that the research was conducted in the absence of any commercial or financial relationships that could be construed as a potential conflict of interest.
